# Additional Effects of Xbox Kinect Training on Upper Limb Function in Chronic Stroke Patients: A Randomized Control Trial

**DOI:** 10.3390/healthcare9030242

**Published:** 2021-02-24

**Authors:** Qurat Ul Ain, Sara Khan, Saad Ilyas, Amna Yaseen, Iqbal Tariq, Tian Liu, Jue Wang

**Affiliations:** 1 The Key Laboratory of Biomedical Information Engineering of Ministry of Education, Institute of Health and Rehabilitation Science, School of Life Science and Technology, Xi’an Jiaotong University, Xi’an 710049, China; qurat.iimc@gmail.com; 2 National Engineering Research Center for Healthcare Devices, Guangzhou 510500, China; 3 The Key Laboratory of Neuro-informatics & Rehabilitation Engineering of Ministry of Civil Affairs, Xi’an 710049, China; 4 Physiotherapy Department, Mukkabir College, Gujrat 50700, Pakistan; sarah.warraich4@gmail.com; 5 Department of Computer Science, Faculty of Information Technology, University of Central Punjab, Lahore 54000, Pakistan; saadkhan1290@gmail.com; 6 Riphah College of Rehabilitation & Allied Health Sciences, Faculty of Rehabilitation and Allied Health Sciences, Riphah International University, Islamabad 46000, Pakistan; amna.yaseen@riphah.edu.pk (A.Y.); iqbal1tariq@gmail.com (I.T.)

**Keywords:** Box and Block Test, Fugl-Meyer Assessment Scale for Upper Extremity, motor function, rehabilitation, stroke, upper extremity, virtual reality, Xbox 360

## Abstract

Background: Xbox Kinect-based virtual reality, being a novel approach, has therapeutic benefits in rehabilitation and its use is encouraged in stroke rehabilitation of upper extremities. Objective: Primary aim of the current study is to investigate the additional effects of Xbox Kinect training in combination with routine physiotherapy exercises based on each component of Fugl-Meyer Assessment Scale for Upper Extremity (FMA-UE). Moreover, effect of upper limb rehabilitation on cognitive functions was also assessed. Methods: This study was a parallel arm randomized control trial. Fifty-six participants were recruited and randomly allocated to either an Xbox Kinect training group (XKGT) or exercise training group (ETG). Measures of concern were recorded using FMA-UE, Box and Block Test (BBT), and Montreal Cognitive Assessment (MOCA). Evaluation was conducted at baseline and after completion of intervention at the sixth week. Results: There were significant differences from pre- to post-intervention scores of FMA-UE and BBT (*p* < 0.001) in both groups, whereas no difference was observed for MOCA (XKTG p value 0.417, ETG p value 0.113). At six-week follow-up there were significant differences between both groups in FMA-UE total score (*p* < 0.001), volitional movement within synergies (*p* < 0.001), wrist (*p* = 0.021), hand (*p* = 0.047), grasp (*p* = 0.006) and coordination/speed (*p* = 0.004), favoring the Xbox Kinect training group. Conclusion: To conclude, results indicate repetitive use of the hemiparetic upper extremity by Xbox Kinect-based upper limb rehabilitation training in addition to conventional therapy has a promising potential to enhance upper limb motor function for stroke patients.

## 1. Introduction

Despite the fact that stroke mortality is continually being reduced in the United States and globally, the number of individuals living with chronic post-stroke symptoms is still rising [1]. This ongoing stroke-related physical disability is primarily related to the common [2] and persistent difficulty in using the upper extremities [3].

In strokes, motor function impairments are more frequent, as brain areas (e.g., primary motor cortex, anterior frontal lobe) being responsible for movement execution and planning are affected [4]. Recent studies also confirmed some functions (e.g., spatial attention, spatial awareness, multisensory integration of feedback) represented in the posterior parietal cortex are also involved in voluntary movements [5]. Moreover, another vital cause interfering with motor task performance after a stroke is the intensity of coupled cognitive impairments [6].

Therefore, the most common consequence of stroke is motor dysfunction. Other impairments include sensory deficits, impaired visual perception, speech dysfunction, swallowing problems and cognitive decline [7]. Stroke patients with upper extremity related functional impairments are more susceptible to difficulties in performing activities of daily life, such as dressing, self-care and eating [7,8].

Motor impairments related to upper extremities include altered muscle tone, impaired motor control, limited range of motion, muscle weakness, laxity or contractures [9]. As activities of daily living (ADLs) and human quality of life are dependent on upper extremity performance, one of the major rehabilitation goals for a stroke patient is improving arm and hand functions and enabling the patient to perform ADLs independently [10]. However, full recovery of upper extremity functions cannot be achieved in most of the survivors [9].

A striking option for improving rehabilitation outcomes is through higher doses in terms of time, repetitions and intensity for every session [11]. However, clinical trials of higher doses have not shown a large amount of progress that can change clinical outcomes, regardless of whether conducted in the early [12] or chronic stage [13,14]. Furthermore, the best stroke rehabilitation therapy for upper limb motor function in outpatients has yet to be determined. A recent Cochrane review for upper extremity functional improvements after stroke concluded that quality evidence is still lacking for superiority of any routinely practiced intervention [12,15].

In accordance with these facts, the need to focus on interventions offering a higher dose and intensity for functional recovery to minimize the stroke-related residual inabilities in chronic stroke patients is crucial [16].

Recently, rapid increases in technology utilization for rehabilitation have been observed. Particularly, virtual reality has emerged as an effective intervention. Virtual reality using exer-gaming played on devices including the Nintendo Wii, PlayStation and Microsoft Xbox 360 Kinect are being utilized as interventions. Video games allow patients to practice activities in a familiar environment, thereby increasing the dose of intervention [17]. Among the recent game systems, Microsoft Xbox 360 Kinect is unique with its advanced camera technology capable of perceiving human body movements sensitively in three dimensions without a remote control or marker [18].

Xbox Kinect training is considered a recent topic in stroke rehabilitation, as the first randomized clinical trial was published in 2013. Since then, a number of studies have been investigating its utilization, yet conclusive outcomes have not yet been developed, necessitating further investigation before adding Xbox Kinect training as part of routine care for stroke rehabilitation of upper extremities [19].

Xbox Kinect is basically a device for playing video games and was not designed as a medical intervention. It recognizes individual movements using infrared camera sensors. It enables the user to see one’s posture and movement on screen and perform actions freely in a real-time virtual reality environment [17]. A number of studies have investigated the outcomes of Xbox Kinect training-based upper extremity rehabilitation in stroke patients [7,18,19,20].

Virtual reality Xbox Kinect is capable of administering an intervention with more repetitions and higher intensity while keeping the patient motivated and engaged, which is thereby helpful in introducing neuroplasticity [7,21,22]. Facilitation of functional neuroplasticity after Xbox Kinect training has also been supported by functional magnetic resonance imaging studies [23,24]. August et al. reported increased activity in the primary and secondary motor cortex, responsible for voluntary motor output. Performing activities of the hand and arm via virtual reality training activates the mirror neuron system which thereby improves functional abilities [25]. These virtual reality-based systems introduce practice-dependent functional enhancements of the impaired limb by facilitating cortical reorganization [26,27]. Another study by Park et al. supported the therapeutic benefits of Xbox Kinect training in stroke rehabilitation [28]. A study conducted by Merians et al. found that virtual reality enhances motion velocity of fingers on the hemiplegic side, strength and range of motion [29]. Moreover, Xbox Kinect training has been found to significantly improve upper arm function along with wrist and hand function [30].

In the current study, we focused on the effect of Xbox Kinect training-based rehabilitation on specific components of the Fugl-Meyer Assessment Scale for Upper Extremity (FMA-UE). In 2019, Jeon and Moon reported results of subscales of FMA-UE, but their sample size was very small. In 2018, Schuster-Anft et al. [31] investigated the effect of virtual reality-based upper limb training for four weeks, but they only used the box and block test as a concordant test, among others.

Recently, a number of studies have investigated the effect of virtual reality on upper limb function after stroke. Laver et al., 2018 [27], in the Cochrane Database of Systematic Reviews, reported about 35 new studies (between 2015 and 2018) observing virtual reality for upper limb function rehabilitation after stroke. In light of these facts, the current study is the first of its kind to explore how FMA-UE subscales can benefit from six weeks of Xbox Kinect training in comparison to time-equivalent conventional therapy alone. Moreover, we also explored the effect of upper limb rehabilitation on cognition.

## 2. Methodology

A parallel arm randomized control trial was conducted in Railway General Hospital, Rawalpindi, Pakistan. The study was completed over six months from February 2019 to July 2019. The study was approved by the ethical committee of Riphah College of Rehabilitation Sciences (Reference number Riphah/RCRS/REC/00514). The clinical trial was registered retrospectively with ClinicalTrials.gov: NCT04669431.

Participants recruited in the trial were stroke outpatients from the neuro-rehabilitation department of the hospital. Both genders within the range of 40–70 years, with their first stroke attack or 6 months or more since their last stroke, and with a score above 4 on the Modified Ashworth Scale (MAS) were included in the study. Those having severe arm or shoulder pain, severe spastic hemiplegia, severe cognitive or visual impairment and patients on drugs that alter functional performance were excluded from the study.

Furthermore, participants were required to be able to read and write their name in the local national language, Urdu, and English. Patients were precisely informed about the procedure and possible risk factors of the study and signed written informed consent forms before participation. The sample size of the study was calculated using an open epi-tool (Open Source Epidemiologic Statistics for Public Health, Version 3.01) [32]. The open epi-tool was accessed from Islamabad, Pakistan in January 2019. Mean and standard deviation for the FMA-UE experimental group was 43.05 ± 12.59. The control group was 34.44 ± 10.53 and a reference study [18] was used for calculating the sample size. The calculated sample size was 58, but due to time constraints we were only able to gather a sample of 56 stroke participants. The sampling technique used was non-probability purposive sampling and the lottery method was used for randomization of the sample into two groups. The measures of concern were assessed using a Modified Ashworth Scale (MAS), Fugl-Meyer Assessment Scale for Upper Extremity (FMA-UE), Box and Block Test (BBT) and Montreal Cognitive Assessment (MOCA). Measurements were recorded at baseline, before initiation of intervention and after 6 weeks, and at the end of intervention, by two blind assessors. Excellent inter-rater as well as intra-rater reliability has been established for FMA-UE (0.995–0.996) [33] as well as the subcomponents of FMA-UE (Synergy 0.983, Wrist 0.994, Hand 0.999, and Coordination 0.988) [34].

### 2.1. Intervention

Participants were given an intervention for six weeks. The experimental group was given Xbox Kinect training-based rehabilitation training for upper extremities along with the conventional exercise therapy, whereas the control group or exercise training group received only conventional exercise therapy for training upper limb function. The duration of intervention was the same for both intervention groups.

#### 2.1.1. Experimental Group (Xbox Kinect Training Group)

A total of 28 participants in this group were trained using Xbox Kinect 360 [7]. Games specifically requiring upper extremity movements were selected from the Kinect adventure pack and Kinect sports pack [20]. For training, the equipment including console, Xbox Kinect, and LED screen were all set up in a dedicated room. The participant was 1.5 to 2 m away from the Kinect sensor. Before initiation of each session, the position of the sensor was adjusted for the patient to ensure maximum motion capture and optimal position. The participants were first given a demonstration of all games and the first week was an orientation week in which patients were told about the procedures and trained for the intervention by a physiotherapist. After the orientation week, for the next two weeks participants played a game named “tennis player,” while in third and fourth weeks, in addition to the tennis player, another game called “joy riding” was also played. Further, in the last two, i.e., fifth and sixth, weeks the participants were trained by playing three games. “Rally ball” was added to the previous two games. All these games were selected on the basis of movement requirements of the upper extremities, while lower extremities were only functional for maintaining a standing position and slight side-to-side movement. Participants in this group also performed conventional training exercises along with Xbox Kinect training for 20 min. The intervention was given for five days a week and each session lasted for 35–40 min.

#### 2.1.2. Control Group (Exercise Training Group)

In this group, 28 participants were trained to improve their upper limb function by performing conventional physical therapy exercises. Each session was given by a trained physiotherapist in the specified cubical of a rehabilitation department. The session was initiated with mild-to-moderate sustained stretching in a pain-free range for 10–30 s. After that, the physiotherapist assisted in attaining a weight bearing position in upper limb extension and this was repeated 1–3 times. The third step included performing different exercises, including tasks related to activities of daily living like basket lifting, folding towels, turning a key into a lock, reaching forward, reaching sideways, ball grasping, picking up small blocks, and lifting cans and pencils, either with assistance of a physiotherapist or just under supervision. Each task was performed for 3–4 min. The time for each physical therapy session was 35–40 min and was given for 5 days a week, for a total duration of six weeks.

### 2.2. Statistical Analysis

The IBM Statistical Package for the Social Sciences version 20 (Armonk, NY, USA, 2011) was used for analysis. Demographic and baseline data were given as mean and standard deviation for both intervention groups. Normality of data was analyzed at baseline and the test application was decided based on the Shapiro-Wilk value. In terms of age, there was no statistically significant difference among both groups. The Montreal Cognitive Scale and Box and Block test scores showed skewed distribution, with a Shapiro-Wilk significance value of 0.003 and 0.014, respectively, so non-parametric tests (Mann-Whitney U Test, Wilcoxon Signed-Rank Test) were applied. The Shapiro-Wilk value for FMA-UE was 0.219, so parametric tests (Independent Samples T-Test and Paired Samples T-Test) were applied for analysis.

## 3. Results

**Figure 1:**
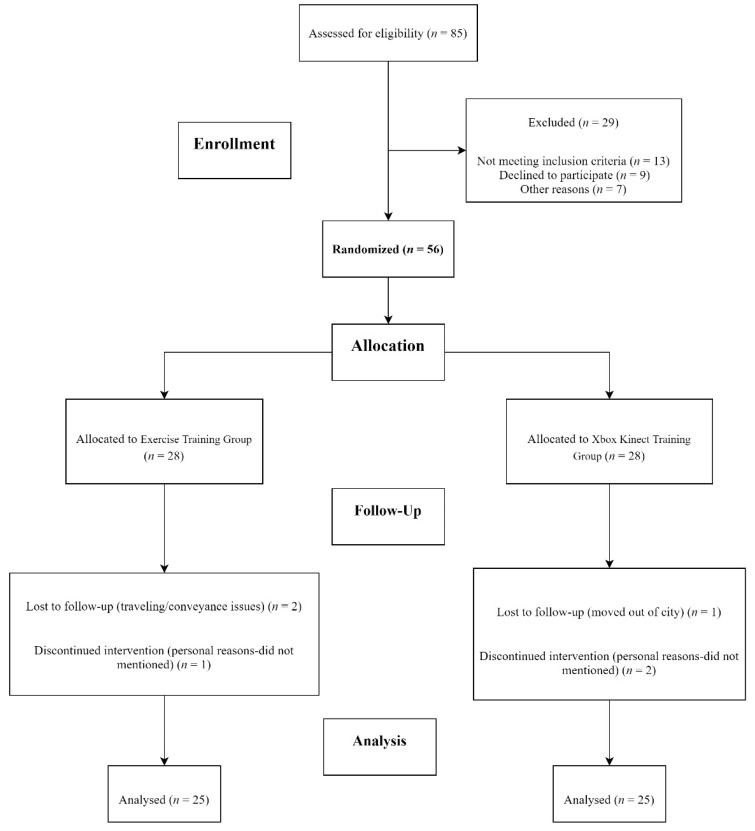
CONSORT 2010 flow diagram.

Around 85 patients were screened for recruitment in the study. Fifty-six patients meeting the set criterion were divided into two groups: the Xbox Kinect training group (*n* = 28) and exercise training group (*n* = 28). There were six dropouts due to personal reasons and transportation issues. The final analysis included 50 participants. A detail of the recruitment and participants analyzed is given in Figure 1.

The participants who completed the study included 43 males and 7 females. Detailed demographics and baseline recordings are mentioned in Table 1.

At post-treatment, FMA-UE showed significant improvements in both groups (*p* < 0.001), however, across group analysis statistically significant improvement was seen in the Xbox Kinect training group (*p* < 0.001). Each component of the FMA-UE was also analyzed, and detailed results are given in Table 2 and Table 3.

As baseline data was not equally distributed among the two groups for the Box and Block test, both for dominant and non-dominant hands, nonparametric tests were applied (Table 4). Across group analysis showed no statistically significant difference in either group (*p* = 0.719 and *p* = 0.076). However, within-group analysis showed statistically significant results in both groups for the dominant hand, as well as the non-dominant hand (*p* < 0.001).

The Montreal Cognitive Scale (MOCA) assessed by the Mann-Whitney U Test (Table 5) for differences across groups post-intervention showed no statistically significant result (*p* = 0.477); likewise, within-group analysis (Wilcoxon Signed-Rank Test) also showed no difference after intervention in the Xbox Kinect training group (*p* = 0.417), as well as the exercise training group (*p* = 0.113).

## 4. Discussion

Gaming systems based on virtual reality help to perform intense, repetitive and targeted movements. Active movement and involvement of the patients also helps to increase the strength of the affected upper extremity [23]. To our knowledge, this is the first study to evaluate additional effects of Xbox Kinect training-based rehabilitation in comparison to conventional exercises on motor recovery of upper extremity in chronic stroke patients by analyzing each component of FMA-UE.

The results of our study showed statistically significant improvement in FMA-UE for the Xbox Kinect training group. However, there was no statistically significant difference for the Box and Block test and Montreal Cognitive Assessment among both groups. FMA-UE showed a statistically significant difference between baseline and post-intervention measurements among the two groups, yet the across-group analysis and mean scores clearly indicate significant improvement in the Xbox Kinect training group. Moreover, looking at the components of FMA-UE, the majority of components showed improvement within the two groups. However, the across-group analysis of effect difference clearly indicates a noticeably significant difference in volitional movement within synergies, volitional movement mixing synergies, hand, grasp and coordination/speed, favoring the Xbox Kinect training group.

Several studies have been conducted to study the effect of Xbox Kinect training. Sin et al. [7] also investigated the effect of additional Xbox Kinect training in chronic stroke patients with a similar duration of intervention (six weeks). This study used similar tools, except for range of motion: FMA-UE and BBT. They also reported significant improvement in FMA-UE, but the result of the BBT contradicts the current study. Another randomized control trial was conducted to evaluate the effect of the Microsoft Xbox 360 Kinect training on upper extremity motor functions for sub-acute stroke patients. Unlike the current study, the intervention was given for four weeks and assessment tools included BBT and FMA-UE. Outcome scores contradict our study, as BBT showed significant improvement after intervention, whereas no difference was observed in FMA-UE in comparison to conventional therapy [18]. Ayhan Askın and colleagues also confirmed additional beneficial effects of Kinect-based virtual reality training in addition to conventional exercise therapy [20]. Recently, a few meta-analyses have also supported the use of virtual reality-based rehabilitation for enhancing upper extremity motor function and quality of life in stroke patients [35,36]. Laver et al., in their Cochrane systematic review, deny the use of interactive video gaming or virtual reality as more beneficial in comparison to conventional therapy, with strong evidence. Yet virtual reality being used as an adjunct therapy to the conventional treatment options may benefit upper limb function and ADLs [27].

Schuster-Anft et al., 2018 also conducted a study to directly compare virtual reality-based training with conventional therapy for four weeks by using BBT as the primary outcome tool. They recorded outcome measures at two-months follow-up and found major improvement in first two weeks of training, with no significant difference among the two groups. In the current study, we recorded measurements only before and after the intervention, but our results contradict the similar effects of both conventional and virtual reality training [31]. Jeon et al. evaluated the effects of four-week training using Xbox Kinect training in sub-acute stroke patients. This study reported not only the effectiveness of Xbox Kinect training in comparison to conventional therapy, but also analyzed the components of FMA-UE and found significantly greater improvements in wrist and hand sub-domains of FMA-UE [30]. In the current study regarding the components of FMA-UE motor function, our results clearly show volitional movement within synergies, hand function—more specifically, mass flexion and extension—and coordination/speed are greatly improved by the virtual reality-based Xbox Kinect training. We aimed to enhance stroke rehabilitation by supporting activation of new motor projection regions and resting synapses, thereby restoring movement. Stimulation of motor activity and movements at various levels may lead to enduring restructuring of the central nervous system [37], and repetitive virtual reality training is also known to activate the motor areas [38], hence improving the functional outcomes of upper extremities.

Studies have shown a decline in upper limb function with a decline in cognitive function [39]. Therefore, another important concern of the current study was to investigate if virtual reality-based upper extremity training has some beneficial effects on cognitive ability or not. Cognition was assessed using the Montreal Cognitive Assessment tool. The results of the current study suggest no change in cognition in response to upper limb functional recovery or virtual reality-based upper limb training. Hyo-Lyun et al. correlated FMA-UE with cognitive ability using the Montreal Cognitive Assessment and mini-mental state examination and found a correlation between FMA-UE and cognition. The study suggests considering cognitive ability while planning rehabilitation sessions for chronic stroke patients [40]. A study conducted by Timothy J. Wolf et al. focused on evaluating the effect of cognitive orientation to occupational performance and found it to be effective in improving both cogitation and upper limb performance [1]. However, in the current study, the intervention was not cognition-oriented and no cognitive training was specifically employed. This might be the reason why there was no change in cognition status. We may infer that declining cognition with upper limb function cannot be reversed directly by improving upper limb function, without specifically focusing on cognition while designing rehabilitation plans.

## 5. Conclusions and Limitations

To conclude, results indicate repetitive use of the hemiparetic upper extremity by Xbox Kinect training-based upper limb rehabilitation training, in addition to conventional therapy, has a promising potential to enhance upper limb motor function for stroke patients. Results are consistent with previous studies and support the use of Xbox Kinect training in rehabilitation settings for chronic stroke patients. Moreover, Xbox Kinect training has prominent effects in enhancing volitional movement within synergies and wrist and hand function, along with coordination, but upper limb rehabilitation not designed specifically for targeting cognitive function has no effect on cognition. In the future, it would be favorable to investigate the effects of Xbox Kinect training with a larger sample size and factors like gender, age, multiple strokes, site of lesion and co-morbidities. There are some aspects that need to be addressed and clarified, such as the dosage of Xbox Kinect routine training and when to incorporate it. Future studies should also focus on developing specially designed games for different types of stroke patients based on their needs. Finally, in order to assess the changes at a CNS level induced by exer-gaming, functional MRI can also be incorporated.

## Figures and Tables

**Table 1:** Demographics and Baseline data.

Variable	Xbox Kinect Training Group	Exercise Training Group
Age (Mean ± SD)	57.48 ± 10.60	57.68 ± 10.43
Gender (Male, Female)	23 (92%), 2 (8%)	20 (76%), 5 (24%)
Modified Ashworth Scale (Mean ± SD)	1.44 ± 1.00	1.56 ± 1.08
Montreal Cognitive Assessment (Mean ± SD)	27.04 ± 2.31	27.00 ± 2.70
Fugl-Meyer Assessment UE (Mean ± SD)	29.16 ± 14.33	26.96 ± 12.44
Box and Block Test Dominant Hand (Mean ± SD)	22.80 ± 15.00	22.48 ± 18.42
Box and Block Test Non-Dominant Hand (Mean ± SD	32.44 ± 13.85	33.96 ± 13.84

**Table 2:** Across group analysis for Fugl-Meyer Assessment Scale for Upper Extremity total score and each component.

Across Group Analysis for Fugl-Meyer Assessment Upper Extremity (FMA-UE)					
Variable	Xbox Kinect Training Group		Exercise Training Group		Effect Size
	Group	Mean ± SD	Mean ± SD	*p*-Value	Partial Eta Squared
FMA-Total Score	Pre	29.16 ± 14.33	26.96 ± 12.45	0.565	
	Post	52.20 ± 10.69	35.36 ± 12.73	<0.001	
Training Effect Difference		23.04 ± 11	8.40 ± 5.48	<0.001	0.425
Reflex Activity	Pre	3.40 ± 1.32	3.36 ± 1.60	0.924	
	Post	3.92 ± 0.40	3.56 ± 0.92	0.78	
Training Effect Difference		0.52 ± 1.41	0.20 ± 1.73	0.478	0.011
Volitional movement within synergies	Pre	8.28 ± 5.47	5.48 ± 3.83	0.042	
	Post	14.08 ± 4.15	6.88 ± 3.89	<0.001	
Training Effect Difference		5.80 ± 5.97	1.40 ± 3.41	0.002	0.175
Volitional movement mixing synergies	Pre	2.84 ± 1.88	2.56 ± 1.73	0.587	
	Post	5.32 ± 2.92	3.92 ± 1.91	0.051	
Training Effect Difference		2.48 ± 2.36	1.36 ± 1.15	0.038	0.086
Volitional movement no synergy	Pre	3.12 ± 2.67	2.56 ± 1.93	0.4	
	Post	4.32 ± 1.90	3.44 ± 1.58	0.082	
Training Effect Difference		1.20 ± 2.91	0.88 ± 1.73	0.64	0.005
FMA-Wrist	Pre	4.72 ± 3.76	3.36 ± 3.31	0.182	
	Post	7.48 ± 2.64	5.44 ± 3.37	0.021	
Training Effect Difference		2.76 ± 2.81	2.08 ± 3.17	0.427	0.013
FMA-Hand	Pre	2.20 ± 1.73	2.20 ± 2.06	1.001	
	Post	3.92 ± 2.46	2.52 ± 2.40	0.047	
Training Effect Difference		1.72 ± 3.11	0.32 ± 2.59	0.05	0.058
FMA-Grasp	Pre	4.96 ± 3.79	4.36 ± 2.77	0.526	
	Post	8.96 ± 3.36	6.08 ± 3.68	0.006	
Training Effect Difference		4.00 ± 2.14	1.72 ± 3.07	0.004	0.162
FMA-Coordination/Speed	Pre	2.88 ± 1.53	2.80 ± 1.75	0.865	
	Post	4.88 ± 1.30	3.48 ± 1.87	0.004	
Training Effect Difference		2.00 ± 2.12	0.68 ± 1.54	0.015	0.116
*N* = 25 Each Group					

FMA = Fugl-Meyer Assessment, FMA-UE = Fugl-Meyer Assessment for Upper Extremity.

**Table 3:** Within group analysis for Fugl-Meyer Assessment Scale for Upper Extremity total score and each component.

Within Group Analysis for Fugl-Meyer Assessment Upper Extremity (FMA-UE)				
Variable	Group	Baseline	Post Intervention	*p*-Value
		Mean ± SD	Mean ± SD	
FMA-Total Score	XKTG	29.16 ± 14.33	52.20 ± 10.69	<0.001
	ETG	26.96 ± 12.45	35.36 ± 12.73	<0.001
Reflex Activity	XKTG	3.40 ± 1.32	3.92 ± 0.40	0.079
	ETG	3.36 ± 1.60	3.56 ± 0.92	0.569
Volitional movement within synergies	XKTG	8.28 ± 5.47	14.08 ± 4.15	<0.001
	ETG	5.48 ± 3.83	6.88 ± 3.89	0.052
Volitional movement mixing synergies	XKTG	2.84 ± 1.88	5.32 ± 2.92	<0.001
	ETG	2.56 ± 1.73	3.92 ± 1.91	<0.001
Volitional movement no synergy	XKTG	3.12 ± 2.67	4.32 ± 1.90	0.051
	ETG	2.56 ± 1.93	3.44 ± 1.58	0.018
FMA-Wrist	XKTG	4.72 ± 3.76	7.48 ± 2.64	<0.001
	ETG	3.36 ± 3.31	5.44 ± 3.37	0.003
FMA-Hand	XKTG	2.20 ± 1.73	3.92 ± 2.46	0.011
	ETG	2.20 ± 2.06	2.52 ± 2.40	0.543
FMA-Grasp	XKTG	4.96 ± 3.79	8.96 ± 3.36	<0.001
	ETG	4.36 ± 2.77	6.08 ± 3.68	0.01
FMA-Coordination/Speed	XKTG	2.88 ± 1.53	4.88 ± 1.30	<0.001
	ETG	2.80 ± 1.75	3.48 ± 1.87	0.038
*N* = 25 Each Group				

FMA = Fugl-Meyer Assessment, XKTG= Xbox Kinect Training Group, ETG = Exercise Training Group.

**Table 4:** Within- and across-group analysis for dominant and non-dominant hand utilizing the Box and Block Test.

**Across Group Analysis for Box and Block Test (BBT)**						
**Variable**	**Group**	**Xbox Kinect Training Group**		**Exercise Training Group**		
		**Mean ± SD**	**Mean Rank**	**Mean ± SD**	**Mean Rank**	***p*-Value**
Dominant Hand	Baseline	22.80 ± 15.00	26.32	22.48 ± 18.42	24.68	0.691
	Post Intervention	40.64 ± 13.03	30.5	31.20 ± 20.82	20.5	0.719
Training Effect Difference		17.84 ± 9.24	33.52	8.72 ± 8.22	17.48	<0.001
Non-Dominant Hand	Baseline	32.44 ± 13.85	24.76	33.96 ± 13.84	26.24	0.015
	Post Intervention	50.28.32 ± 17.69	29.16	42.20 ± 15.56	21.84	0.076
Training Effect Difference		17.84 ± 9.64	33.4	8.24 ± 4.53	17.6	<0.001
W**ithin Group Analysis for Box and Block Test (BBT)**						
**Variable**	**Group**	**Baseline**	**Post Int**	**Mean Rank (+)**	**Mean Rank (−)**	***p*** **-value**
		**Mean ± SD**	**Mean ± SD**			
Dominant Hand	Xbox Kinect Training Group	22.80 ± 15.00	40.64 ± 13.03	13	0	<0.001
	Exercise Training Group	22.48 ± 18.42	31.20 ± 20.82	13	0	<0.001
Non-Dominant Hand	Xbox Kinect Training Group	32.44 ± 13.85	50.28.32 ± 17.69	13	0	<0.001
	Exercise Training Group	33.96 ± 13.84	42.20 ± 15.56	13	0	<0.001
*N* = 25 Each Group						

**Table 5:** Within- and across-group analysis for Montreal Cognitive Assessment (MOCA).

**Across Group Analysis for Montreal Cognitive Assessment (MOCA)**					
**Variable**	**Xbox Kinect Training Group**		**Exercise Training Group**		
	**Mean ± SD**	**Mean Rank**	**Mean ± SD**	**Mean Rank**	***p*** **-Value**
Baseline	27.04 ± 2.32	25.2	27.00 ± 2.69	25.8	0.883
Post Intervention	27.32 ± 1.75	24.06	27.32 ± 2.87	26.94	0.477
Training Effect Difference	0.28 ± 1.88	23.5	0.32 ± 0.90	27.5	0.293
**Within Group Analysis for Montreal Cognitive Assessment (MOCA)**					
**Treatment Groups**	**Baseline**	**Post Int**	**Mean Rank (+)**	**Mean Rank (−)**	***p*** **-value**
	**Mean ± SD**	**Mean ± SD**			
Xbox Kinect Training Group	27.04 ± 2.32	27.32 ± 1.75	7	5.8	0.417
Exercise Training Group	27.00 ± 2.69	27.32 ± 2.87	6.09	12	0.113
*N* = 25 Each Group					

## Data Availability

The datasets used and/or analyzed during the current study are available from the corresponding author on reasonable request.

## References

[1] Wolf T.J., Polatajko H., Baum C., Rios J., Cirone D., Doherty M., McEwen S. (2016). Combined cognitive-strategy and task-specific training affects cognition and upper-extremity function in subacute stroke: An exploratory randomized con-trolled trial. Am. J. Occup. Ther..

[2] Licher S., Darweesh S.K.L., Wolters F.J., Fani L., Heshmatollah A., Mutlu U., Koudstaal P.J., Heeringa J., Leening M.J.G., Ikram M.K. (2019). Lifetime risk of common neurological diseases in the elderly population. J. Neurol. Neurosurg. Psychiatry.

[3] Ward N.S., Brander F., Kelly K. (2019). Intensive upper limb neurorehabilitation in chronic stroke: Outcomes from the Queen Square programme. J. Neurol. Neurosurg. Psychiatry.

[4] Teka W.W., Hamade K.C., Barnett W.H., Kim T., Markin S.N., Rybak I.A., Molkov Y.I. (2017). From the motor cortex to the movement and back again. PLoS ONE.

[5] Xu G.-Q., Lan Y., Zhang Q., Liu D.-X., He X.-F., Lin T. (2016). 1-Hz Repetitive Transcranial Magnetic Stimulation over the Posterior Parietal Cortex Modulates Spatial Attention. Front. Hum. Neurosci..

[6] Dennis A., Bosnell R., Dawes H., Howells K., Cockburn J., Kischka U., Matthews P., Johansen-Berg H. (2011). Cognitive Context Determines Dorsal Premotor Cortical Activity During Hand Movement in Patients After Stroke. Stroke.

[7] Sin H., Lee G. (2013). Additional Virtual Reality Training Using Xbox Kinect in Stroke Survivors with Hemiplegia. Am. J. Phys. Med. Rehabil..

[8] Kiper P., Szczudlik A., Agostini M., Opara J., Nowobilski R., Ventura L., Tonin P., Turolla A. (2018). Virtual Reality for Upper Limb Rehabilitation in Subacute and Chronic Stroke: A Randomized Controlled Trial. Arch. Phys. Med. Rehabil..

[9] Lang C.E., Bland M.D., Bailey R.R., Schaefer S.Y., Birkenmeier R.L. (2013). Assessment of upper extremity impairment, function, and activity after stroke: Foundations for clinical decision making. J. Hand Ther..

[10] Simonetti D., Zollo L., Milighetti S., Miccinilli S., Bravi M., Ranieri F., Magrone G., Guglielmelli E., Di Lazzaro V., Sterzi S. (2017). Literature Review on the Effects of tDCS Coupled with Robotic Therapy in Post Stroke Upper Limb Rehabilitation. Front. Hum. Neurosci..

[11] Lohse K.R., Lang C.E., Boyd L.A. (2014). Is More Better? Using Metadata to Explore Dose–Response Relationships in Stroke Rehabilitation. Stroke.

[12] Winstein C.J., Wolf S.L., Dromerick A.W., Lane C.J., Nelsen M.A., Lewthwaite R., Cen S.Y., Azen S.P. (2016). Effect of a task-oriented rehabilitation program on upper extremity recovery following motor stroke: The ICARE randomized clinical trial. JAMA.

[13] Lang C.E., Strube M.J., Bland M.D., Waddell K.J., Cherry-Allen K.M., Nudo R.J., Dromerick A.W., Birkenmeier R.L. (2016). Dose response of task-specific upper limb training in people at least 6 months poststroke: A phase II, single-blind, randomized, controlled trial. Ann. Neurol..

[14] Klamroth-Marganska V., Blanco J., Campen K., Curt A., Dietz V., Ettlin T., Felder M., Fellinghauer B., Guidali M., Kollmar A. (2014). Three-dimensional, task-specific robot therapy of the arm after stroke: A multicentre, parallel-group randomised trial. Lancet Neurol..

[15] Pollock A., Farmer S.E., Brady M.C., Langhorne P., Mead G.E., Mehrholz J., Van Wijck F. (2014). Interventions for improving upper limb function after stroke. Cochrane Database Syst. Rev..

[16] Lang C.E., Macdonald J.R., Gnip C. (2007). Counting Repetitions: An Observational Study of Outpatient Therapy for People with Hemiparesis Post-Stroke. J. Neurol. Phys. Ther..

[17] Lee G. (2013). Effects of Training Using Video Games on the Muscle Strength, Muscle Tone, and Activities of Daily Living of Chronic Stroke Patients. J. Phys. Ther. Sci..

[18] Afsar S.I., Mirzayev I., Yemisci O.U., Saracgil S.N.C. (2018). Virtual Reality in Upper Extremity Rehabilitation of Stroke Patients: A Randomized Controlled Trial. J. Stroke Cerebrovasc. Dis..

[19] Xavier-Rocha T.B., Carneiro L., Martins G.C., Vilela-Júnior G.D.B., Passos R.P., Pupe C.C.B., do Nascimento O.J.M., Haikal D.S.A., Monteiro-Junior R.S. (2020). The Xbox/Kinect use in poststroke reha-bilitation settings: A systematic review. ARQ Neuropsiquiatr..

[20] Aşkın A., Atar E., Koçyiğit H., Tosun A. (2018). Effects of Kinect-based virtual reality game training on upper extremity motor recovery in chronic stroke. Somatosens. Mot. Res..

[21] Chang Y.-J., Chen S.-F., Huang J.-D. (2011). A Kinect-based system for physical rehabilitation: A pilot study for young adults with motor disabilities. Res. Dev. Disabil..

[22] Johnson L., Bird M.-L., Muthalib M., Teo W.-P. (2018). Innovative STRoke Interactive Virtual thErapy (STRIVE) online platform for community-dwelling stroke survivors: A randomised controlled trial protocol. BMJ Open.

[23] Bao X., Mao Y., Lin Q., Qiu Y., Chen S., Li L., Cates R.S., Zhou S., Huang D. (2013). Mechanism of Kinect-based virtual reality training for motor functional recovery of upper limbs after subacute stroke. Neural Regen. Res..

[24] You S.H., Jang S.H., Kim Y.-H., Hallett M., Ahn S.H., Kwon Y.-H., Kim J.H., Lee M.Y.L. (2005). Virtual reality–induced cortical reorganization and associated locomotor recovery in chronic stroke: An experimenter-blind randomized study. Stroke.

[25] August K., Lewis J.A., Chandar G., Merians A., Biswal B., Adamovich S. analysis of neural mechanisms underlying rehabilitation in virtual reality: Activating secondary motor areas. Proceedings of the 2006 International Conference of the IEEE Engineering in Medicine and Biology Society, IEEE.

[26] Saposnik G., Levin M., Outcome Research Canada (SORCan) Working Group (2011). Virtual reality in stroke rehabilitation: A meta-analysis and implications for clinicians. Stroke.

[27] Laver E.K., Lange B., George S., Deutsch E.J., Saposnik G., Crotty M. (2017). Virtual reality for stroke rehabilitation. Cochrane Database Syst. Rev..

[28] Park D.-S., Lee D.-G., Lee K., Lee G. (2017). Effects of virtual reality training using Xbox Kinect on motor function in stroke sur-vivors: A preliminary study. J. Stroke Cerebrovasc. Dis..

[29] Merians A.S., Poizner H., Boian R., Burdea G., Adamovich S. (2006). Sensorimotor Training in a Virtual Reality Environment: Does It Improve Functional Recovery Poststroke?. Neurorehabilit. Neural Repair.

[30] Kwon J.-S., Park M.-J., Yoon I.-J., Park S.-H. (2019). Effects of virtual reality training on upper extremity function and activities of daily living in patients with sub-acute stroke: A double-blind randomized clinical trial. NeuroRehabilitation.

[31] Schuster-Amft C., Eng K., Suica Z., Thaler I., Signer S., Lehmann I., Schmid L., McCaskey M.A., Hawkins M., Verra M.L. (2018). Effect of a four-week virtual reality-based training versus conventional therapy on upper limb motor function after stroke: A multicenter parallel group randomized trial. PLoS ONE.

[32] Dean A.G., Sullivan K.M., Soe M.M. (2019). OpenEpi: Open Source Epidemiologic Statistics for Public Health.

[33] Gladstone D.J., Danells C.J., Black S.E. (2002). The Fugl-Meyer Assessment of Motor Recovery after Stroke: A Critical Review of Its Measurement Properties. Neurorehabilit. Neural Repair.

[34] Duncan P.W., Propst M., Nelson S.G. (1983). Reliability of the Fugl-Meyer Assessment of Sensorimotor Recovery Following Cerebrovascular Accident. Phys. Ther..

[35] Domínguez-Téllez P., Moral-Muñoz J.A., Salazar A., Casado-Fernández E., Lucena-Antón D. (2020). Game-Based Virtual Reality Interventions to Improve Upper Limb Motor Function and Quality of Life After Stroke: Systematic Review and Meta-analysis. Games Heal. J..

[36] Karamians R., Proffitt R., Kline D., Gauthier L.V. (2020). Effectiveness of virtual reality-and gaming-based interventions for upper extremity rehabilitation poststroke: A meta-analysis. Arch. Phys. Med. Rehabil..

[37] Pascual-Leone A., Amedi A., Fregni F., Merabet L.B. (2005). The plastic human brain cortex. Annu. Rev. Neurosci..

[38] Keller J., Štětkářová I., Macri V., Kühn S., Pětioký J., Gualeni S., Simmons С.D., Arthanat S., Zilber P. (2020). Virtual reality-based treatment for regaining upper extremity function induces cortex grey matter changes in persons with acquired brain injury. J. Neuroeng. Rehabil..

[39] Rand D. (2018). Proprioception deficits in chronic stroke—Upper extremity function and daily living. PLoS ONE.

[40] Roh H.-L., Kim C.-W. (2019). Cognition and Upper-extremity Function Influence on Performance of Activities of Daily Living in Patients with Chronic Stroke. J. Korean Soc. Phys. Med..

